# Behavioral diversity as a potential positive indicator of animal welfare in bottlenose dolphins

**DOI:** 10.1371/journal.pone.0253113

**Published:** 2021-08-30

**Authors:** Lance J. Miller, Lisa K. Lauderdale, Jocelyn L. Bryant, Jill D. Mellen, Michael T. Walsh, Douglas A. Granger

**Affiliations:** 1 Conservation Science and Animal Welfare Research, Chicago Zoological Society–Brookfield Zoo, Brookfield, Illinois, United States of America; 2 Biology Department, Portland State University, Portland, Oregon, United States of America; 3 Department of Comparative, Diagnostic & Population Medicine, College of Veterinary Medicine, University of Florida, Gainesville, Florida, United States of America; 4 Institute for Interdisciplinary Salivary Bioscience Research, University of California, Irvine, California, United States of America; Institute of Deep-sea Science and Engineering, Chinese Academy of Sciences, CHINA

## Abstract

Accredited zoological facilities are committed to fully understanding the behavioral, mental, and physical needs of each species to continuously improve the welfare of the animals under their professional care and detect when welfare has diminished. In order to accomplish this goal, internally consistent and externally valid indicators of animal welfare are necessary to advance our understanding of the current welfare status of individual animals. Historically, efforts have focused on monitoring visible or observable signs of poor health or problem behavior, but lack of signs or problems does not necessarily demonstrate that an individual animal is thriving. The current study examined fecal hormone metabolite levels and behavior for two species of bottlenose dolphins (*Tursiops truncatus* and *Tursiops aduncus*) from 25 different accredited zoological facilities. At the time of the study, all facilities were accredited by the Alliance of Marine Mammal Parks and Aquariums and/or the Association of Zoos and Aquariums. This was part of the multi-institutional study ‘Towards understanding of the welfare of cetaceans in zoos and aquariums” commonly referred to as the Cetacean Welfare Study. Behavioral diversity was calculated using the Shannon Diversity Index on species-appropriate behavioral events. Behavioral diversity was compared to the fecal metabolites of cortisol, aldosterone, and the ratio of cortisol to dehydroepiandrosterone (DHEA) as well as the stereotypic behavior of route tracing. Similar to previous studies on other species, there was a significant inverse relationship between behavioral diversity and both fecal cortisol metabolites and route tracing. Additionally, a significant inverse relationship also exists between behavioral diversity and the ratio of fecal cortisol to DHEA metabolites. Behavioral diversity and fecal aldosterone metabolites were not associated. Additional research is still needed to validate behavioral diversity as an indicator of positive animal welfare for bottlenose dolphins and across species. However, based on current results, facilities could utilize behavioral diversity combined with other measures of welfare to more comprehensively evaluate the welfare of bottlenose dolphins.

## Introduction

Historically, the field of animal welfare has focused predominantly on negative indicators of animal welfare such as stereotypic behavior [1]. However, absence of a negative indicator of animal welfare does not suggest that an individual animal is thriving [2, 3]. As accredited zoos and aquariums are committed to continuous improvement, it is critical that staff have tools to accurately and reliably assess the welfare of their animals from a positive perspective. This makes it critical to validate positive indicators of animal welfare to ensure that zoological facilities can be successful in meeting their goals of ensuring high levels of animal welfare.

One potential positive indicator of welfare is behavioral diversity [4]. Behavioral diversity can be defined as the frequency and richness of species-typical behavior exhibited by an individual animal [5]. The underlying theory is that if an animal has high behavioral diversity (i.e., frequently exhibits a variety of species-appropriate behaviors), there is an increased likelihood that the animal is experiencing positive welfare. Alternatively, if behavioral diversity is low, the animal is likely stereotyping or lethargic, both typical signs that an animal should be examined for compromised welfare [3, 6]. While behavioral diversity has not been validated as a positive indicator of welfare, research on behavioral restriction and studies on behavioral diversity are starting to provide some evidence that behavioral diversity might hold promise in assessing animal welfare.

When an animal’s behavior is restricted, especially behaviors they are highly motivated to perform, its welfare may be compromised (e.g., mink [7]; pigs [8]; horses [9]; and mice [10]). For example, when mink are denied access to a swimming pool they demonstrate increased cortisol levels similar to levels observed during food deprivation [7]. While having high behavioral diversity does not ensure an animal’s behavioral needs are being met, it does increase the likelihood that an individual is engaged in species-appropriate behaviors they are motivated to perform.

Additionally, several studies have examined behavioral diversity as it relates to other indicators of animal welfare. The majority of studies examining the relationship between behavioral diversity and stereotypic behavior have found an inverse relationship (e.g., sows [11]; laboratory rabbits [12]; small felids [13]; and songbirds [14]). The only exception was one study where behavioral diversity was not statistically related to varying levels of stereotypic behavior in two species of bears [15]. In addition, an inverse relationship was found between behavioral diversity and fecal cortisol in cheetahs [5] as well as chimpanzees [16]. While cortisol production and acute activation of the hypothalamic-pituitary-adrenal (HPA) axis may be beneficial to an animal, repeated or chronic activation can lead to hypercortisolism and hypocortisolism which can have damaging effects on welfare [17, 18]. Thus, the inverse relationship between behavioral diversity and fecal cortisol metabolites might provide further support that behavioral diversity may be an index that demonstrates the ability of a zoological facility to meet important behavioral needs of individual animals.

While previous research has examined the relationship between behavioral diversity and fecal cortisol metabolites [5, 16], there have been no efforts, to our knowledge, examining the relationship with aldosterone or the ratio of cortisol to dehydroepiandrosterone (DHEA). Aldosterone is another marker of adrenal activity that can increase during potential stressors such as environmental changes or physical challenges [19]. Previous research has suggested that aldosterone can be another marker used for cetaceans to give a multifaceted approach to assessing adrenal activity [20, 21]. Cortisol:DHEA on the other hand, has primarily been utilized with humans where elevated ratios typically are a sign of adrenal fatigue, depression, or illness [22]. However, it has also been examined as a marker of welfare in other species (e.g., rhesus macaques [23]; domestic cattle [24]; pigs [25]). DHEA is another product of the HPA axis and can impact mental health and immune function [26, 27]. Examination of Cortisol:DHEA in cetaceans would be necessary to better understand the significance as a potential indicator of animal welfare.

As a species that is behaviorally diverse in the wild and adaptable to their environment [28–30], behavioral diversity could also potentially be a positive indicator of welfare for bottlenose dolphins. In the wild, dolphins spend a good proportion of their time engaged in a variety of different behaviors especially while foraging [28–30]. Foraging behaviors can include fluke-in dives and fast swims in order to obtain prey [29, 31, 32]. As a social species, dolphins are also often seen engaged in behaviors such as fluke-out dives, group social balls, engaging in tactile behavior, and interacting with conspecifics [31, 33]. Finally, while the exact meaning of the behaviors is likely context dependent, dolphins can often be seen engaged in a variety of aerial behaviors such as jumps, breaches, and porpoising [34, 35]. Overall, dolphins display a wide variety of behaviors in the wild, and incorporating a variety of these measures into a metric of behavioral diversity, may provide a positive indicator of welfare for dolphins to demonstrate the ability to meet their behavior needs under professional care.

The goal of personnel in accredited zoos and aquariums is to monitor and understand the significance of a variety of behaviors for the dolphins under their professional care. These can include common species-appropriate behaviors as well as less common stereotypic or abnormal behaviors [36, 37]. An example of a stereotypic behavior observed in dolphins is route tracing. The stereotypic behavior of route tracing involves an individual animal swimming in an identical path in a fixed repetitive pattern. This behavior is different from pattern swimming with conspecifics which may include swimming in circles or ovoid patterns. Route tracing is more rigid and less flexible in nature [36, 38]. Dolphins are obligate swimmers and if there is variability to the swimming patterns, the behavior observed would not be considered a stereotypic behavior. An inverse relationship observed between route tracing and behavioral diversity may provide additional support for behavioral diversity as a positive indicator of animal welfare.

Dolphin behavioral diversity under professional care would likely depend on a variety of habitat characteristics and animal management techniques. For example, one could hypothesize that animals that receive enrichment, training, and are in appropriate social groupings would have higher behavioral diversity. Previous research has shown that bottlenose dolphins have significantly higher levels of behavioral diversity immediately following dolphin presentations and interaction programs [36]. These programs utilize positive reinforcement training which is thought to be a beneficial experience for the animals [39, 40]. In addition, these programs may provide interactions and aspects of play that are also thought to be beneficial to individual animals. If behavioral diversity is higher following an experience thought to be positive, this provides some additional evidence that behavioral diversity may be a positive indicator of welfare for bottlenose dolphins.

The goal of the current study was to examine the relationship between behavioral diversity and both behavioral and physiological measures of welfare in bottlenose dolphins. Information gained from this study could provide support for another positive indicator of animal welfare for bottlenose dolphins.

## Materials and methods

### Ethics statement

Approval to conduct this research was given by animal care and veterinary staff at each participating facility. In addition, the study was reviewed and approved by the U.S. Navy Marine Mammal Program Institutional Animal Care and Use Committee #123–2017.

### Subjects and facilities

This study is one part of the larger study entitled “Towards Understanding the Welfare of Cetaceans in Zoos and Aquariums.” Zoos, aquariums, and marine parks that were accredited by the Alliance of Marine Mammal Parks and Aquariums (AMMPA) or the Association of Zoos and Aquariums (AZA) were invited to participate in this portion of the project if they professionally managed at least one of the two focal species. The two subspecies included common bottlenose dolphins (*Tursiops truncatus*) and Indo-Pacific bottlenose dolphins (*Tursiops aduncus*). A semi-random, counterbalanced sampling design was utilized to select two animals from each participating facility trying to create a balanced representation of the study population across the variability of exhibit types, enrichment and training programs constrained by sex and age. The final potential participants included 86 dolphins from 40 different facilities. There were six dolphins observed during the first five-week data collection period that were not observed during the second five-week data collection period. There were eight dolphins observed during the second data collection period that were not observed during the first data collection period and two dolphins were observed during both data collection periods but changed facilities in-between the two data collection periods. S1 Appendix highlights all dolphins that were observed including sex, age, facility id, and minutes visible during observations.

### Data collection

#### Behavioral data

All data were collected between July and November 2018 (first five-week data collection period) and January through April of 2019 (second five-week data collection period). During each of the five weeks, focal animals were videotaped by animal care staff or interns at each facility during one of three time periods. These included morning (8:00–11:00), mid-day (11:00–14:00) and afternoon (14:00–17:00) local time. Animal care staff or interns were requested to film the animals in order to ensure accurate identification of the focal animal. Table 1 displays the video schedule during the study. Video could be taken anytime during the assigned time period as long as it was not within 20 minutes before or after a training session, research session, dolphin presentation or interaction program. In order to habituate the dolphins to the individual with the camera when filming, the videographer stood in the filming location at each facility during scheduled times for a week prior to actual data collection. The videographer was also instructed to avoid interacting (eye contact or engaging with) with the animals during observation periods. Before recording, the videographer held up a piece of paper that included an exhibit code, dolphin name, identification number, observation number, date, and time. Each observation lasted for 25 minutes; observations were conducted three times per week during the month for a total of 375 minutes per five-week data collection period for each focal animal throughout the study. All videos were recorded using a Fuji Film XP120 waterproof video camera fitted with polarized film to reduce glare. All video was recorded above water to ensure videos were comparable across facilities as not all facilities have underwater viewing.

**Table 1:** Observation schedule for video taping focal animals.

Week	Time Period	Monday	Tuesday	Wednesday	Thursday	Friday	Saturday	Sunday
1	8:00–11:00	D1			D2			
	11:00–14:00	D2		D1				
	14:00–17:00			D2	D1			
2	8:00–11:00	D2					D1	
	11:00–14:00						D2	D1
	14:00–17:00	D1						D2
3	8:00–11:00			D1			D2	
	11:00–14:00			D2	D1			
	14:00–17:00				D2		D1	
4	8:00–11:00			D2				D1
	11:00–14:00	D1						D2
	14:00–17:00	D2		D1				
5	8:00–11:00				D1			D2
	11:00–14:00				D2		D1	
	14:00–17:00						D2	D1

Note: D1 is Focal Dolphin 1 and D2 is Focal Dolphin 2.

Videos for the study were scored by nine reliable trained observers after inter-observer reliability was r > 0.80 for event behaviors. This included training observers on the important distinction between pattern swimming within conspecifics and the stereotypic behavior of route tracing. Observers used continuous sampling of behavioral events to count the total number of occurrences of specific behavioral events. The ethogram used for the larger study included more behaviors; however, only species-appropriate behaviors were used to calculate behavioral diversity [16]. Behaviors typically associated with negative welfare such as stereotypic or abnormal behavior, and behaviors that could be associated with lethargy were excluded from the diversity index [4]. Behavioral diversity was calculated using behaviors including fluke-in dive, fluke-out dive, fast swim, group social ball, interact with object, jump/breach, mount, interact with conspecifics, porpoise, spyhop, tactile/rub, and ventral swim (Table 2). Additionally, route tracing was also recorded as a form of stereotypic behavior to be compared to behavioral diversity. All behavioral definitions were adapted from previous studies [29, 36, 41–46].

**Table 2:** Ethogram utilized to score videos by trained reliable observers.

Behavior	Definition
Fast Swim	Dolphin sustains a rapid speed, swimming in one direction, for more than 3 s, producing a wake at the surface (while not chasing another individual).
Fluke-In Dive	Dolphin surfaces and then dives down under the water with the fluke remaining below the surface of the water.
Fluke-Out Dive	Dolphin surfaces and then dives down under the water raising its fluke up in the air and out of the water.
Group Social Ball	Two or more dolphins swim around each other, often mouthing and chasing each other. This is often associated with sexual play. It is extremely difficult to identify the individual behaviors that each animal is doing.
Interact with Conspecific	Dolphin orients toward and mutually interacts with one or more conspecifics for more than 3 s.
Interact with Object	Dolphin interacts with an object which can include holding, carrying, balancing, or pushing the object; interactions will only be counted once if within 3 s of the previous interaction.
Jump/Breach	A large aerial locomotion in which all of the dolphin’s body comes completely out of the water.
Mount	One dolphin’s genital area touches another’s genital area.
Porpoise	Small bows usually performed several times in a row characterized by small forward motion leaps out of the water. The dolphin’s head re-enter the water as the tail is exiting the water.
Spy Hop	Dolphin moves in such a way that the upper part of the body rises above the water in a vertical position.
Tactile/Rub	Dolphin contacts or actively rubs another dolphin a manner that is not considered sexual contact.
Ventral Swim	Dolphin swims inverted with ventral side pointing towards the surface for more than 3 s.
Route Tracing	Dolphin swims in fixed repetitive pattern using the same path to move from one point to another. Dolphin must complete the pattern three or more times to be recorded.

All events were converted to rates by dividing by the total number of minutes visible. Behavioral diversity was then calculated using the Shannon Diversity Index (H) where p is the rate of each behavior observed divided by the total rates of all behaviors observed, ln is the natural log, and ∑ is the sum across behaviors [47]. The Shannon Diversity Index was chosen due to the ability to detect subtle changes in all factors (behaviors) when one factor (behavior) is dominant [48].


$$H=-\sum_{i=1}^{s}p_{i}\ln p_{i}$$

Although a polarized filter was added to each camera, there were instances where animals were not visible for extended periods of time due to water surface glare. In addition, facilities that were seaside had animals that were not visible for extended periods of time due to increases in turbidity due to animal activity, normal water characteristics, or wave action stirring up sediment. Because of these factors, any dolphin without at least 240 minutes visible during one of the five-week periods was dropped from further analysis. Dolphins that had more than 240 minutes in both five-week periods had the second five-week period dropped from analysis; otherwise the data collection period with more than 240 minutes was retained. The reason data from the second five-week data collection period with greater than 240 minutes were dropped was due to the statistical analysis that would exclude rows for any missing data. Data were selected from a single five-week data collection period as dolphins without matching data in both time periods would have been excluded entirely when examining statistical models, further reducing sample size. This process resulted in largest sample size possible while ensuring high quality data. A chi-square test of significance and an independent t-test were used to ensure resulting samples were not significantly different from original sample based on habitat type, sex, or age. All behavioral variables for each individual were averaged across the five-week period for statistical analysis to explore across individuals as opposed to within individual variation.

### Physiological data

All data were collected between July and September 2018 and January through March of 2019. During the same five weeks as video recording at each facility, one fecal sample per focal animal was collected each week for a total of five samples per animal during each five-week data collection period. In order to reduce the variability in hormone concentrations due to diurnal patterns, staff were instructed to collect samples during the last two hours of the work day, generally between 16:00 and 18:00. Collecting samples later in the day also allowed for a larger fecal sample due to fish consumed throughout the day. Samples were collected using a clean disinfected catheter and staff wore disposable gloves to prevent contamination of samples with exogenous hormones from the skin. Samples were transferred directly to a pre-labeled 5ml vial using sterile water if the sample was too viscus. Samples were then immediately frozen at -20°C until shipped on dry ice to the Chicago Zoological Society Endocrine Laboratory for analysis.

Fecal samples were lyophilized at -50°C for 48 hours in a Labconco Freeze Dry System (Labconco model #A65412906 with Edwards oil mist filter #EMF10) and manually pulverized. Samples were then weighed to retain between 0.25g ± 0.005g of homogenized sample and placed into 2.5mL polypropylene screw cap tubes until further analysis. Any samples weighing less than 0.02 g after being lyophilized were excluded from further analysis to avoid the effects of low sample mass on hormone concentrations [48, 49].

Fecal hormone assays were conducted based on previously established protocols with minor modifications [49, 50]. Fecal hormone metabolites were extracted using 80% ethanol in dH20 at a ratio of 2.5 mL per 0.1g of dried sample. Samples were then vortexed and placed on a shaker overnight (Labline Maxi Rotator cat # 4631). The following morning, samples were centrifuged (Fisher Marathon 3000R) at 2500 rpm for 20 minutes. Approximately 200uL of supernatant from each sample was transferred to a new tube and run immediately or stored at -20°C until analysis. Concentrations of fecal cortisol, aldosterone, and DHEA metabolites were measured using commercially available kits from Arbor Assays (cortisol, catalog no. K003-H5; aldosterone, catalog no. KO52-H5) and Genway BioTech (DHEA, catalog no. GWB-719A7E). All assays were validated biochemically and variability between assays was monitored using high and low controls. Biochemical validation consisted of demonstrating parallelism with the standard curve and determining the percentage of exogenous hormone measured through recovery. Samples were run in duplicate on each assay and fecal hormone metabolites were measured by following the manufacturer’s directions. Assay plates were read using a Dynex Technologies MRX Microplate Reader at 405nm. All concentrations are reported as ng/g dry weight and hormone metabolite concentrations for each individual were averaged across the five-week period for statistical analysis. A complete overview of the methods and assay validation techniques can be found by referring to Miller et. al., [51].

### Statistical analysis

Given the non-normal distribution of the physiological data, all relationships were examined using generalized estimating equations (GEE) in SPSS Version 27. GEE can be used when data are not normally distributed and does not require transformations which can make interpretation of results more straightforward [52, 53]. Specifically, the relationship between behavioral diversity and cortisol, aldosterone, and cortisol:DHEA fecal metabolites, as well as route tracing were examined for significance. For all models, the individual dolphin was used as the unit of analysis while controlling for facility, age, and sex. Results were considered significant at p < 0.05.

## Results

Out of the 86 total possible subjects, 47 dolphins at 25 facilities met criteria for a minimum of 240 minutes visible during one of the sampling periods to be included in the final analysis. This included 28 males and 19 females ranging from 4 to 47 years old at the start of the study (average 19.68 years ± 12.39 SD). There were no significant differences based on sex (χ2(1, N = 133) = 0.01, *p* > 0.05), age (t (131) = -1.105, *p* > 0.05), or habitat type (χ2(1, N = 133) = 3.016, *p* > 0.05) between original and resulting samples. This included 43 *Tursiops truncatus* (91.3%) and 4 *Tursiops aduncus* (8.7%). Across all individuals, behavioral diversity ranged from 0.11 to 1.45, fecal cortisol metabolites ranged from 16.76 ng/g to 534.59 ng/g, fecal aldosterone metabolites ranged from 6.04 ng/g to 125.54 ng/g, and fecal cortisol:DHEA metabolites ranged from 0.02 to 0.52, and route tracing ranged from a rate of 0.00 to 0.03 per minute visible. Table 3 summarizes the results of the GEE examining the relationships between behavioral diversity and the behavioral and physiological measures. Overall, with significance values below 0.05, there was an inverse relationship observed between behavioral diversity and both fecal cortisol and cortisol:DHEA metabolites, as well as route tracing when controlling for facility, sex, and age.

**Table 3:** Results from generalized estimating equations examining the relationship between behavioral diversity and hormone levels in bottlenose dolphins.

Variable		Behavioral Diversity
Fecal Cortisol (ng/g)	β	-0.002
	95% CI	-0.003–0.000
	*p*-value	0.013*
Fecal Aldosterone (ng/g)	β	0.000
	95% CI	-0.002–0.002
	*p*-value	0.886
Fecal Cortisol:DHEA	β	-0.957
	95% CI	-1.612 - -0.302
	*p*-value	0.004*
Route Tracing	β	-7.664
	95% CI	-13.393 - -1.935
	*p*-value	0.009*

Note: All values shown are calculated using GEE while controlling for sex and facility; DHEA also measured as ng/g

* *p* < 0.05.

## Discussion

Results from the current study support the idea that behavioral diversity may be a positive indicator of animal welfare for bottlenose dolphins. With an inverse relationship between behavioral diversity and both fecal cortisol and cortisol:DHEA metabolites, and a stereotypic behavior (route tracing), this adds further support to the idea that the animals may be experiencing positive welfare when species-specific behavioral diversity is high. While there is still more research necessary to better understand the role of behavioral diversity as a potential positive indicator of welfare, the current study adds additional evidence for behavioral diversity to be used as a tool to assess welfare.

Similar to previous research with cheetahs [5] and chimpanzees [16], in the current study there was an inverse relationship between fecal cortisol metabolites and behavioral diversity. Previous research has demonstrated that when behavior that an animal is highly motivated to perform is restricted, there can be an increase in cortisol [7]. The inverse relationship observed in the current study as well as previous research would support the idea that behavioral diversity may be a measure of the likelihood animals are experiencing positive welfare. Additionally, the inverse relationship between behavioral diversity and route tracing is similar to the typical inverse relationship observed between behavioral diversity and stereotypic behavior in other taxa [11–14]. It is important to note that swimming in a circular pattern due to habitat shape does not constitute route tracing and having an operational definition and scientific methodology to quantify stereotypic behavior is important when examining animal behavior.

In addition to the inverse relationship with fecal cortisol metabolites and route tracing, behavioral diversity also was significantly related to the ratio of cortisol to DHEA fecal metabolites. As previously noted, the ratio has primarily been used to examine the status of humans and elevated ratios are typically a sign of adrenal fatigue, depression, or illness [22]. However, the cortisol:DHEA has been used successfully in macaques, cattle, and pigs to examine emotional state [23–25]. The relationship observed in the current study provides some support that the ratio may be an indicator of welfare for bottlenose dolphins. However, additional research would be necessary to determine the value of this measure moving forward.

In the current study, there was no significant relationship observed between behavioral diversity and fecal aldosterone metabolites. The lack of significant relationship may suggest aldosterone is not an indicator of welfare for bottlenose dolphins. Alternatively, it could be that the assay used in the current study was not picking up the metabolites necessary to demonstrate changes in fecal aldosterone metabolites. Additionally, it is possible that fecal samples may not be ideal for examining aldosterone in bottlenose dolphins or that the assay is not ideal with fecal samples. Previous research has shown that during a stress test, no significant differences in fecal aldosterone were observed, but serum aldosterone was significantly higher during the stress test for bottlenose dolphins [20]. Additional research is necessary to determine if fecal samples are the best biological sample for examining changes in aldosterone metabolites for bottlenose dolphins.

The current study is part of the largest multi-institutional study examining behavioral diversity as a potential indictor of animal welfare for any species. In short, the expression of high levels of species-specific behavioral diversity may be a positive indicator of animal welfare for bottlenose dolphins. However, the low beta value for the relationship between fecal cortisol metabolites and behavioral diversity and the lack of information on cortisol:DHEA in bottlenose dolphins suggests more research is necessary. However, with the additional finding of the inverse relationship with route tracing, there is enough evidence that behavioral diversity may be a potential positive indicator of animal welfare. With further validation, facilities could utilize behavioral diversity along with other measures of welfare to determine an animal’s current welfare status and identify ways to continually enhance welfare. As it is generally considered best practice in the field to utilize multiple indicators of welfare, behavioral diversity should be paired with other measures of welfare when monitoring animals. One potential limitation of the study was the smaller number of *Tursiops aduncus* and inability to control for subspecies. However, given the similarities between *T*. *truncatus* and *T*. *aduncus*, as well as controlling for facility in the analysis, this was not a major concern. Future research should help ensure there are no species differences in regards to behavioral diversity with bottlenose dolphins. Additionally, as dolphins are homeotherms, it would be recommended that future studies examining physiological indicators should also include water temperature as a covariate in analysis.

Future research priorities should examine factors that lead to higher behavioral diversity and promote the welfare of individual animals as well as research to better understand the physiological measures of welfare used in this study. The results examining the relationship between behavioral diversity and cortisol:DHEA are promising, but clearly additional research is necessary to understand if cortisol:DHEA is biologically relevant for bottlenose dolphins. While overall current evidence would suggest behavioral diversity is a positive indicator of animal welfare, we would encourage additional investigation and evaluation to validate it across a diversity of species with similar sample sizes where possible.

## Supporting information

S1 Data Descriptive Statistics_Miller_et_al_Behavioral Diversity. (XLSX)

S1 Appendix Miller_et_al_Behavioral Diversity. (XLSX)
